# One-year extended Glasgow Outcome Scale and hospital mortality predictors in patients with severe traumatic brain injury in Brazil

**DOI:** 10.1186/cc12275

**Published:** 2013-03-19

**Authors:** R Turon, FR Ferreira, D Prado, P Kurtz, M Damasceno

**Affiliations:** 1 Hospital de Clínicas de Niteroi, Brazil; 2 Clinica São Vicente, Rio de Janeiro, Brazil

## Introduction

Traumatic brain injury (TBI) is a leading cause of death and disability worldwide. Because mortality seriously underestimates the impact of TBI, an outcome tool including vegetative state and severe disability should be used. Our purpose was to study the long-term outcome of a small cohort of severe TBI patients in Brazil.

## Methods

This was a retrospective analysis of a prospectively collected database. We included 34 consecutive adult patients admitted with severe TBI to a tertiary private hospital in Rio de Janeiro, Brazil between 2009 and 2011. We analyzed data on demographics, admission, clinical scores, imaging and complications, as well as hospital mortality and 1-year extended Glasgow Outcome Scale (eGOS) of all patients.

## Results

We analyzed 33 patients with severe TBI. Mean age was 36 (± 20) years and 79% were male. All patients were mechanically ventilated and 70% underwent ICP monitoring. Overall hospital mortality was 36% and 60% had unfavorable outcome (eGOS <4) after 1 year. In univariate analysis, higher APACHE II scores, the presence of midriasis and intracranial hypertension were more frequent in nonsurvivors compared with survivors. Furthermore, older age, higher APACHE II score and worse GCS on admission were associated with unfavorable outcome after 1 year, as measured by eGOS <4.

## Conclusion

Our descriptive results suggest a significant burden of severe TBI in a small cohort of young patients. Moreover, severity of the primary disease, age and pupil reflexes, as well as intracranial hypertension seemed to be associated with worse neurological outcome.

